# OLFACTORY IMPAIRMENT AND MICROSTRUCTURAL INTEGRITY OF THE BRAIN IN THE ARIC STUDY

**DOI:** 10.1093/geroni/igac059.604

**Published:** 2022-12-20

**Authors:** Srishti Shrestha, Xiaoqian Zhu, Kevin Sullivan, Beverly Gwen Windham, Jennifer Deal, Honglei Chen, Michael Griswold, Thomas Mosley

**Affiliations:** University of Mississippi Medical Center, Jackson, Mississippi, United States; University of Mississippi Medical Center, Jackson, Mississippi, United States; UMMC-The MIND Center, Jackson, Mississippi, United States; UMMC-The MIND Center, Jackson, Mississippi, United States; Johns Hopkins University, Baltimore, Maryland, United States; College of Human Medicine, Michigan State University, East Lansing, Michigan, United States; The MIND Center at UMMC, Jackson, Mississippi, United States; UMMC-The MIND Center, Jackson, Mississippi, United States

## Abstract

We examined cross-sectional associations between microstructural integrity of the brain and olfaction in 1417 participants from the ARIC Study who completed MRI scans in 2011-2013 (mean age=76±2 years, 41% male). Microstructural integrity was measured by two diffusion tensor imaging measures, fractional anisotropy (FA, higher=better) and mean diffusivity (MD, higher=worse), and olfaction by a 12-item odor-identification test. In multivariable linear regression models, higher FA in several regions was associated with better olfaction, with the strongest association in the stria terminalis [β:0.333 (95%CI:0.188, 0.478) per standard deviation (SD) higher FA]. Higher MD was associated with lower olfaction in almost all regions, but associations were strongest for some temporal sub-regions [for example, hippocampus, β:-0.796 (95%CI: -0.942, -0.651) per SD higher MD]. Our findings suggest that neuronal microstructural integrity is an important predictor of olfaction; this may also have important implications in understanding early dementia neuropathology as olfaction is affected very early in dementia.

